# The Catalytic Reactivity of Alloys; Ethanol and Formic Acid Decomposition on Cu–Pd(110)

**DOI:** 10.1021/acs.jpcc.2c04881

**Published:** 2022-09-12

**Authors:** Michael Bowker, Richard Holroyd, Neil Perkins

**Affiliations:** † Catalysis Hub, RCAH, Rutherford Appleton Laboratory, Harwell Oxford Campus, Didcot OX11 0QX, United Kingdom; ‡ Max Planck- Cardiff Centre on the Fundamentals of Heterogeneous Catalysis FUNCAT, Cardiff Catalysis Institute, School of Chemistry, Cardiff University, Main Building, Park Place, Cardiff CF10 3AT, United Kingdom; § Chemistry Department, University of Reading, Reading RG6 6AH, United Kingdom; ∥ Element Six, Campus, Harwell, Fermi Avenue, Didcot OX11 0QR, United Kingdom

## Abstract

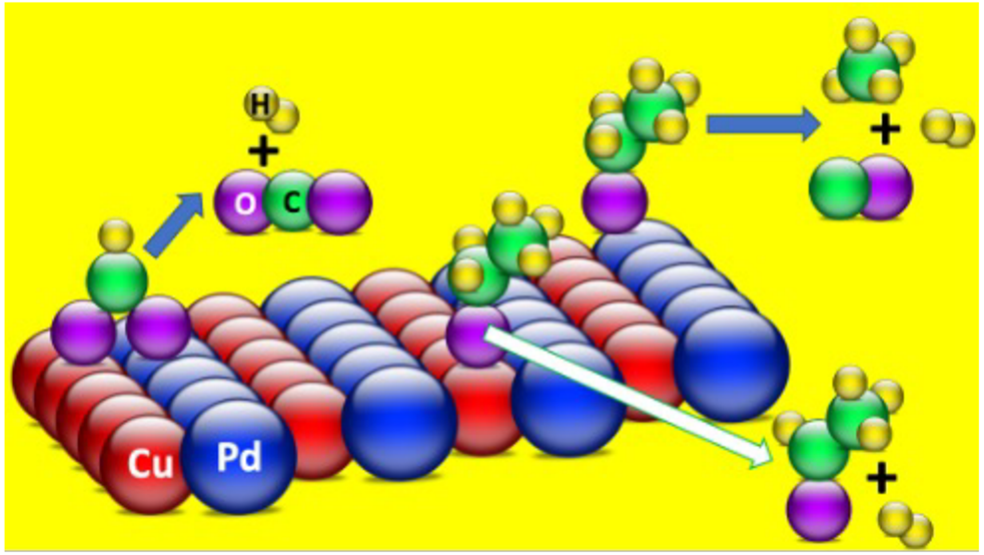

The effect of alloying Cu and Pd on the reactivity pattern for formic acid and for ethanol has been examined. The electronic structure of the material is strongly affected by the alloying, with the d-band lowered in energy and filled, compared with Pd alone. Hence the reactivity would be expected to be strongly affected by the alloying. This appears to be the case for formic acid decomposition, whose decomposition temperature in temperature-programmed desorption is shifted by alloying and is between the temperatures for the individual components (at 350 K, compared with 250 and 470 K for Pd and Cu, respectively). However, when a different molecule is chosen as the probe of surface reactivity, namely, ethanol, we come to a very different conclusion. Here the individual reactivity patterns for the two elemental components of the alloy are seen, namely, dehydrogenation on the Cu (to produce acetaldehyde) and decarbonylation on Pd (to methane and CO). There are effects of alloying on destabilizing the former pathway and stabilizing the latter, but the major conclusion from this work is that it is not average electronic structure that dictates reactivity but the individual atomic nature of the surface components. Only monodentate adsorbates truly probe this behavior.

## Introduction

A perennial problem in catalysis has been the establishment of the relative importance of ligand (or electronic) versus ensemble (or geometric) effects on metal reactivity. Here we will show that, for an alloy surface comprising both Cu and Pd atoms in a well-ordered arrangement, what determines the surface chemistry is predominantly *the chemical nature of the individual atoms*. This contrasts with the fact that this system is a near-ideal alloy, with good atom mixing and an exothermic enthalpy of mixing of 0.17 eV/atom^1^ and with greatly changed electronic structure compared with the component metals.^2−7^ The d-band is shifted away from the Fermi level compared with pure Pd, implying filling of the d-band, but is closer to the Fermi level than for pure Cu.

**Figure 1:**
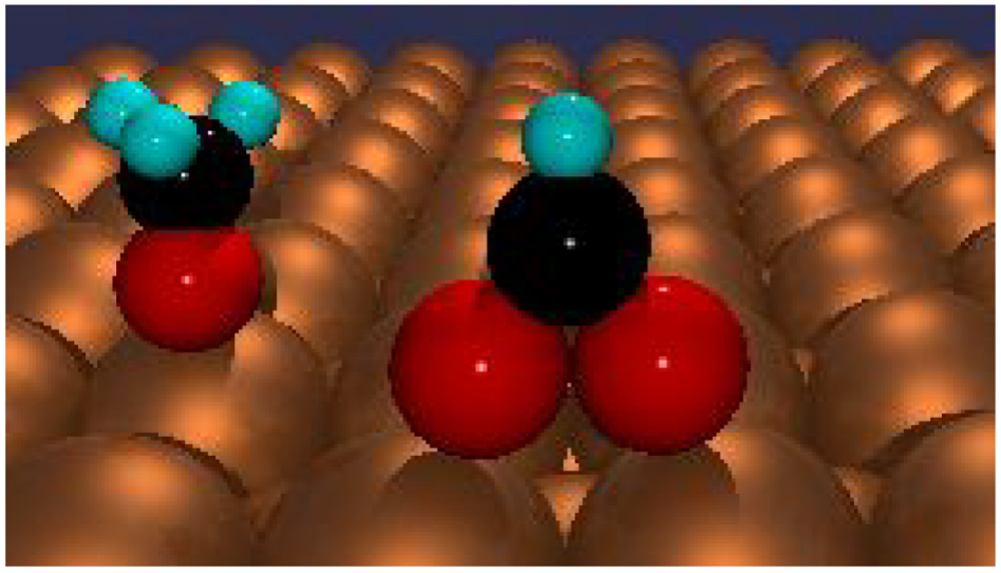
Schematic models of alkoxy and formate bonding on a Cu surface. Reproduced from Bowker.^11^

The probe molecules we have used to illustrate this contention are two quite different entities, namely, a simple alcohol, ethanol, and the simplest carboxylic acid, formic acid. The results from the two molecules are very different in terms of their reactivity modification by alloying. However, we contend that this only relates to the fact that ethanol forms an alkoxy species as an intermediate, singly bonded to atoms in the surface through the oxygen atom,^8−10^ whereas the acid is bidentate, bonding as a formate species to two adjacent surface atoms; see schematic Figure 1.^11^ The latter has been shown to be the case on monometallic Cu(110), for instance, with both oxygen atoms appearing to be in identical environments^12−16^ and in a number of other recent publications for a variety of Cu surfaces, from single crystals of different orientation^17−20^ to catalysts.^21,22^

**Figure 2:**
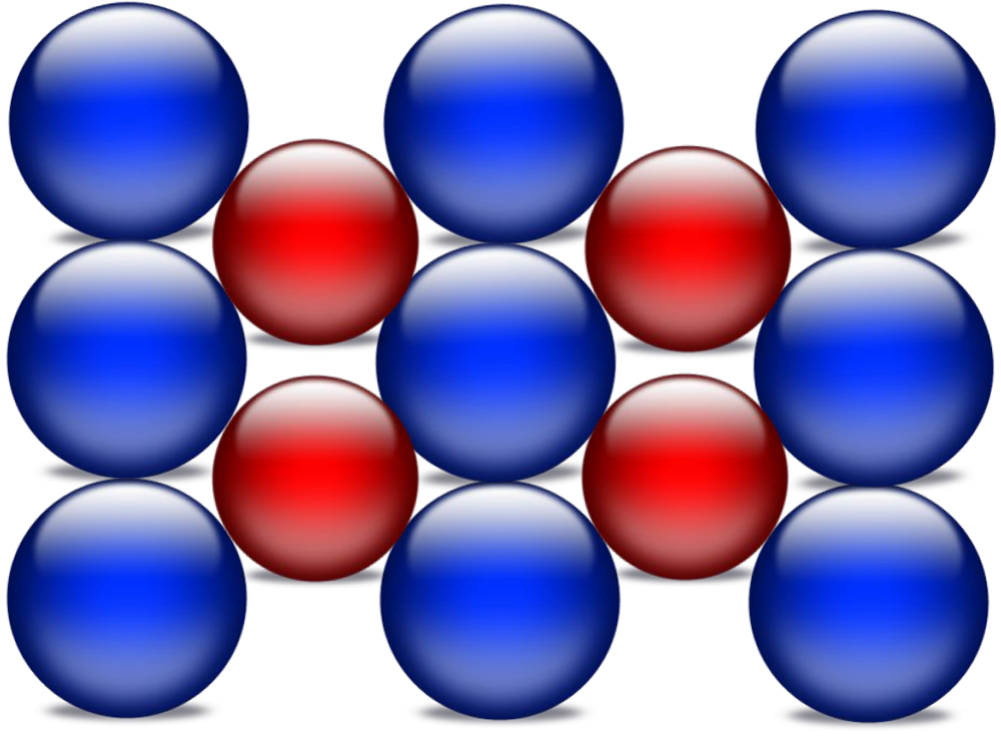
Structure of the 1:1 CuPd(110) surface. Cu atoms in red, Pd in blue.

We have carried out kinetic and surface analysis studies on well-defined Cu–Pd alloys,^23−26^ as have others.^3,5,27−33^ The 1:1 CuPd(110) surface used here is a body-centered cubic structure, and the surface structure is illustrated in Figure 2, as described by a number of authors.^6,38^ The preparation of such a surface needs care, as detailed by Laboda-Cackovic et al.,^27−31^ including avoidance of the order–disorder transition of the sample, which occurs above 770 K, which could incur loss of the sample integrity. The surface consists of a nearly close-packed unit cell with one metal atom central and the other metal comprising the corners of the cell, so that a variety of sites of Pd–Pd, Cu–Cu, and Pd–Cu are available. Details of the preparation are given below and in the Supporting Information.

Here we intend only to highlight the effects of alloying on reactivity, manifested mainly in temperature-programmed desorption (TPD) experiments with ethanol and formic acid. The question we wish to address is the following—does alloying produce a homogeneous surface of quite different reactivity from the individual atoms or a surface for which adjacent atoms are modified by the second alloying metal, or is the reactivity just a mix of the two properties?

## Methods

The experiments were carried out in a ultrahigh-vacuum (UHV) system described in the Supporting Information but, most importantly for this paper, containing facilities for dosing of gases into the vacuum system and for temperature-programmed desorption and molecular beam measurements,^34^ using mass spectrometry. This machine operated at a base pressure of 2 × 10^–10^ Torr and was equipped with Auger, low energy electron diffraction (LEED), and a mass spectrometer for TPD and molecular beam measurements. The crystal was mounted on 0.15 mm diameter tungsten wires, suspended between two 2 mm tungsten support rods on the manipulator, and the sample could be cooled to 90 K and heated to the desired treatment temperature, measured by an attached thermocouple. It was cleaned, following the methods of Laboda-Cackovic et al.,^27−31^ by cycles of argon ion bombardment (current density 2–3 μA cm^–2^) followed by annealing to 750 K and monitoring of the Cu/Pd Auger ratio. Checks of top surface layer composition were also done by following CO TPD spectra. More details about the treatments can be found in the Supporting Information.

## Results and Discussion

**Figure 3:**
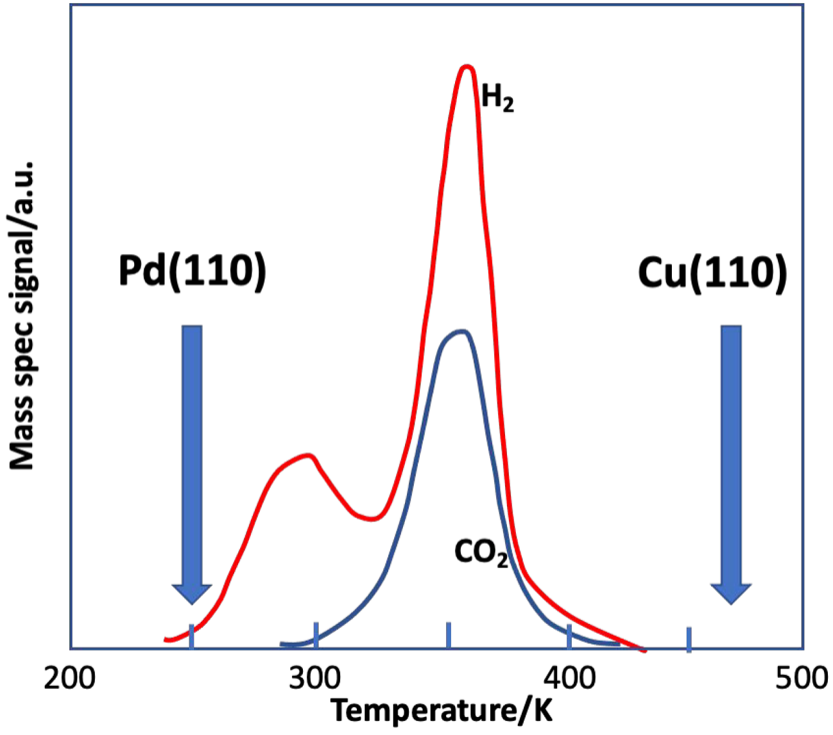
Desorption after adsorbing formic acid on the CuPd(110) surface, showing formate decomposition at 350 K, which yields coincident desorption of H_2_ and CO_2_. Also illustrated are the peak decomposition temperatures for Pd(110)^36^ and Cu(110).^34,35^

Formic acid adsorbs dissociatively at this temperature on Cu(110)^12,34−36^ and Pd(110)^37^ resulting in the production of a formate species on the surface. Figure 3 shows the result for TPD after adsorption on the alloy surface at 185 K, and the peak decomposition temperatures for Cu(110)^12,34−36^ and Pd(110)^37^ are also shown. The first peak of hydrogen evolution at 280 K is due to the recombination of H atoms, which are dissociated from the formic acid at lower temperature. The coincident desorption of H_2_ and CO_2_ at 350 K is rate-limited by the formate decomposition, not by the desorption energy of CO_2_ or H_2_, which, as individual adsorbates, desorb from the surface at lower temperatures. This TPD indicates that the alloy is homogeneous, since the formate has very different stability, intermediate between that of the component metals and has only one state; it is much reduced in stability compared with Cu(110)^36^ (by −120 K) and much increased in stability compared with Pd(110)^37^ (by +100 K). This agrees with the calculations of Yuan and Zhang who also find that the heat of adsorption of formate on this surface is intermediate between those on Cu and Pd.^38^ This is then also in line with the changed electronic structure, which is intermediate, though perhaps more like Cu, in that the d-band is filled.^1−5^ Furthermore, there is only one desorption state. Thus, these findings appear to support the idea of a new material whose different electronic structure leads to the very different stability seen in Figure 3. However, in what follows we will show that this is very misleading by using a more definitive probe molecule.

**Figure 4:**
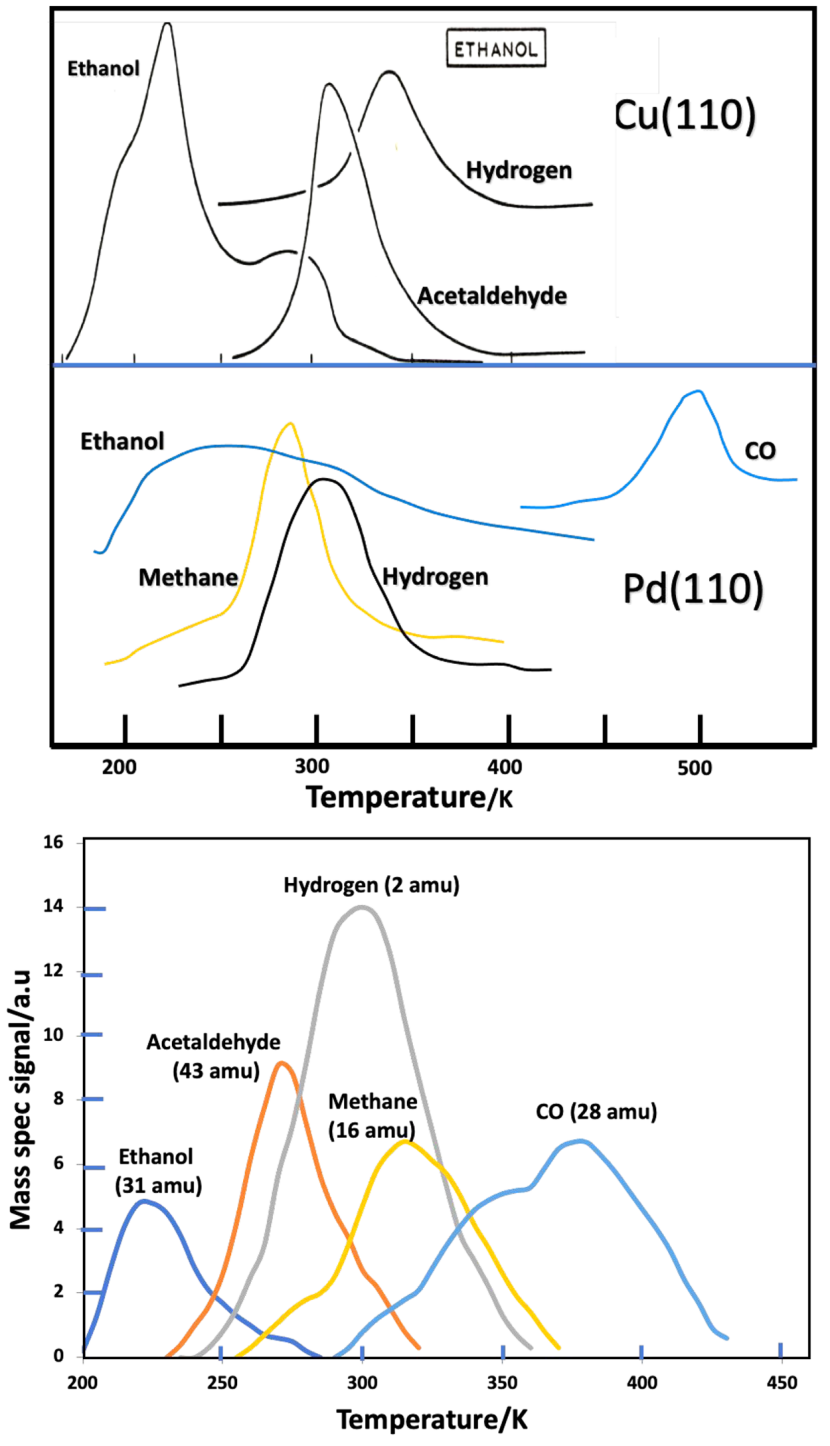
(a) Ethanol TPD from Pd and Cu, showing the decomposition products and very different reaction pathways. The figure is compiled from data for Pd(110)^36^ and for Cu(110).^35^ (b) TPD after adsorption of ethanol on the Cu–Pd alloy surface at 180 K. The product pattern is generally similar to that from the two individual components of the alloy, showing both dehydrogenation (ethanal desorption at 270 K) and decarbonylation (methane desorption at 310 K).

Figure 4a shows the desorption and decomposition of ethanol from Pd(110)^39,40^ and Cu(110),^9^ these reactions having been reported in detail previously and are only shown here for comparison with the results for the alloy surface shown in Figure 4b. The important features to note in Figure 4a are

1. Cu shows only the dehydrogenation of ethanol, as reported originally by Madix and co-workers^8−10^ producing acetaldehyde and hydrogen from the surface in a very selective manner; there are no other products. The desorption temperature of the acetaldehyde is ∼310 K.
2. Pd shows only decarbonylation, yielding methane and CO as carbon-containing products into the gas phase in a very selective manner. The desorption temperature for the methane is ∼290 K.^39,40^

Figure 4b shows the desorption from the Cu–Pd(110) alloy surface. It is quite clear that this looks like a combination of the desorption from the monometallic surfaces shown above; that is, it displays both the dehydrogenation typical of Cu and the decarbonylation typical of Pd, even though these elements are intimately mixed at the surface. Thus, it appears that the individual surface atoms behave in a way typical of the element, and we can state that *individual atomic character dominates the reactivity* for such unidentate molecules. Alloying effects are secondary, but real. The most notable effect is perhaps on the CO desorption, which is shifted to lower temperature by ∼100 K compared to Pd(110) as shown in Figure 4b and is at a similar peak temperature to that found by Hammoudeh et al. for CO adsorption alone^27^ and as suggested by theory;^5^ it is much more strongly held than on Cu, where desorption occurs typically at ∼200 K.^41^ A similar shift is found by Jeroro et al.^42^ for CO adsorption on 0.5 ML of Cu deposited on Pd(111) and also from methanol decomposition on that surface. In Figure 3b the methane peak is shifted to higher temperature (by ca. 40 K), while the acetaldehyde desorption is shifted to lower temperature (by ca. −40 K), corresponding with changes in activation energy for the decomposition of the surface intermediates, which produce them of only ∼9 kJmol^–1^ or roughly 10% of the reaction activation energy (using the Redhead equation^43^). These shifts are in the direction that might intuitively be expected; that is, Pd is alloyed with Cu, and so the intermediate that produces methane (the methyl group^39,40^) is stabilized by the presence of the less reactive metal, and the peak is shifted to higher temperature. On the other hand, Cu is alloyed with Pd, and so the ethoxy intermediate is destabilized by the more reactive metal, dehydrogenation to acetaldehyde proceeds at a higher rate, and hence the peak appears at a lower temperature than for the pure Cu metal.

**Figure 5:**
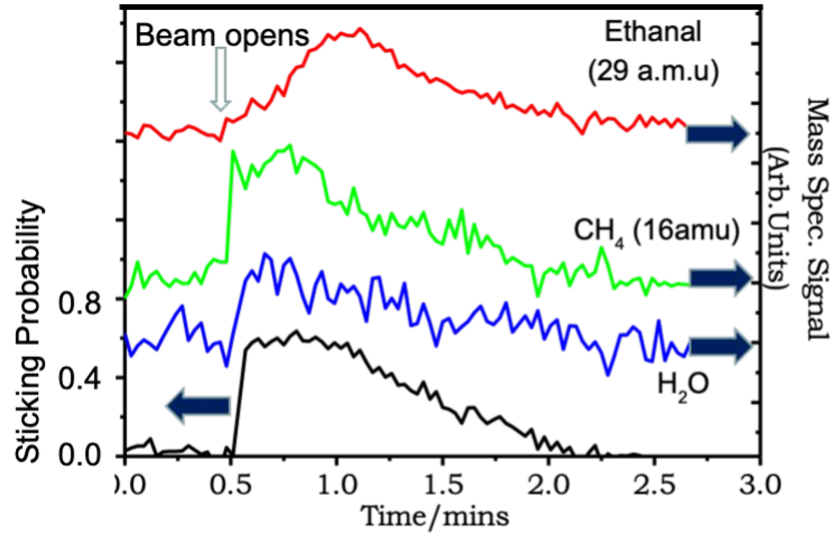
Molecular beam raw reactor data for ethanol in the beam reacting with oxygen predosed CuPd surface at 343 K surface temperature. The beam is opened to hit the surface at 0.5 min.

This combined behavior is also seen in thermal molecular beam experiments, where both products are seen to evolve from the surface (the details of these measurements are given in the Supporting Information). In this case we had to predose the crystal with oxygen to observe measurable sticking of the ethanol molecule on the surface (i.e., greater than ∼0.02). As can be seen in Figure 5 the sticking probability is hugely increased by the predosed oxygen atoms to ∼0.6. Both methane and acetaldehyde desorb into the gas phase if the reaction is carried out above 310 K. At this reaction temperature, 343 K, both products, methane and acetaldehyde, can desorb, but CO is left adsorbed on the surface. So, the drop in sticking and product formation is a combination of removal of preadsorbed oxygen (as water) and blockage of the surface by CO. Although both products desorb it might be expected that acetaldehyde would evolve first (from the TPD of Figure 4b), but clearly methane evolves first. That it evolves immediately is not a surprise, since 343 K is above the decomposition temperature peak, but why is acetaldehyde delayed? This is possibly due to a higher local sticking probability of ethanol at the Pd sites. However, more likely is that, for some reason, the Cu sites are effectively blocked by the presence of the oxygen, and some support for this can be seen in Figure 5, since ethanol begins to evolve and peaks after the water evolution peak, which appears to closely follow the methane evolution.

The TPD result for ethanol is somewhat surprising since it is well-known that Cu–Pd represents one of those alloy systems where the electronic structure is strongly modified by the alloying.^1−7,44^ Thus, the alloy appears to be group 11-like in nature; that is, there is low electron density at the Fermi level typical of those elements, and the d-electron states are positioned below the Fermi level. This certainly appears very different from the situation for Pd itself and might be taken to imply some charge transfer to d-states of the Pd, as shown by a number of authors. However, it must be noted that Weightman et al. have proposed net charge transfer to be very low between Cu and Pd and that most of the electronic structure change is intra-atomic in nature, due to s → d transfer within the Pd valence levels.^45^

An important development to describe surface reactivity has been the d-band model developed by Norskov et al.,^46−48^ for which the position of the d-band with, respect to the Fermi level, and the bandwidth are considered to be major descriptors for alloy reactivity, as espoused also by earlier workers in catalysis (see, e.g., Dowden^49^). The data presented here appear to contrast with what we may predict from such models, since clearly the d-band is significantly altered compared with either individual metal, whereas the identity of the reactive behavior of those components is essentially maintained. Thus, we consider that the ensemble effect, rather than a ligand effect, dominates the reactivity here. It must be noted that, for some other systems too, the d-band model does not seem to apply well, as described further below. Norskov and Hammer themselves suggest that the ligand effect band model fails for certain classes of alloys, including CuPd alloys.^50^

Why do we claim that the ensemble effect is important, rather than the electronic effects? The evidence from formic acid decomposition appears to counter this proposal; in particular, the stability of the formate, seen in Figure 3, is almost exactly between that of the two metals, it is very much shifted in peak temperature from the individual metals, and there appears to be only one type of formate. However, this seems not to be due to an electronic effect, as shown above for the ethoxy, but is probably due either to the fact that the two oxygen atoms in the formate bond to two different metals—one to the Cu and one to the Pd—or that the transition state involves heteroatom interaction. Yuan et al.^38^ calculate similar binding energies for bidentate formate on this CuPd surface for Pd–Pd, Pd–Cu, and Cu–Cu, but with higher binding to the latter (−2.54 eV) compared with Pd–Pd (−2.47 eV). This is a difference of only ∼7 kJ/mol. Note that it has already been shown earlier by scanning tunnelling microscopy (STM) that under these conditions of treatment (see methodology in the Supporting Information) this is an ordered alloy with alternating Cu and Pd^23,24^ in the surface layer, although this was for a thin-layer material that was produced by depositing Pd onto Cu(110). It would be very useful to have a detailed study of a bulk CuPd crystal carried out by high-resolution STM, especially to resolve the exact binding sites of ethoxy and formate species, but such bulk alloy materials are not easy to obtain and require the detailed preparation methods described in the Supporting Information. Alternatively well-ordered thin films can be produced, but most workers currently focus on single-site catalysts (e.g., dilute layers of Pd in Cu^32^), and again, well-ordered mixed layers can be rather difficult to achieve in that way. Note that Ji et al.^51^ have also used aberration-corrected high-angle annular dark-field scanning transmission electron microscopy (HAADF-STEM) to atomically resolve the surface on large CuPd nanoparticles of the same body-centered cubic (bcc) bulk structure as used here.

The ensemble then has very different stability for the intermediate since the two elements are involved in formate bonding. Further support for this view comes from our earlier work on an alloy with much lower Pd content. In that case the surface structure of the alloy is different (with a lower bulk Pd content of only 15%); the surface layer is completely Cu,^25,26,52,53^ and we should have identified only electronic effects due to the high level of Pd that is present in the second layer (half a monolayer in a p(2 × 1) structure), to which every surface Cu atom is attached. The shift in formate stability was very small: it was destabilized compared with Cu alone, but only by ∼17 K,^52^ because it is only bonded to surface Cu atoms, and the electronic effect from the adjacent sublayer Pd is therefore evidently very small, if it is present at all, since the lattice is slightly expanded compared with Cu and may be the entire cause of this small difference. Similarly, working with dilute Pd/Cu catalysts Qiu et al.^54^ found that the effect of alloying on Pd was mainly geometric, with little evidence of an electronic component. Jeroro et al.^42^ compared the behavior of PdCu and PdZn and, in contrast to the latter alloy, found little evidence of an electronic effect on adsorption of CO and methanol, considering that the changes that were seen were almost entirely due to ensemble effects. Further Qiu et al.^54^ examined the effect of alloying on CO_2_ hydrogenation (reverse water gas shift mainly) on CuPd alloys and concluded that changes in behavior were dominated by geometric, ensemble effects.

We note, however, that the effect on CO desorption is rather marked. The CO is much more strongly held than on Cu and more weakly than on Pd. However, we would contend that this again is due to the fact that CO binds multiply to the surface and therefore bonds to both Cu and Pd. The exact binding site is not known for this alloy, but it is interesting to note that, for CO adsorption on a mixed-alloy thin-layer material, Hager et al.^33^ conclude that both ligand and electronic effects play a role. However, Sakong et al.^6^ consider that changes in CO binding are mainly due to changes in the nature of the Pd ensembles available where threefold Pd sites bind strongly, but when diluted by Cu they are weakened by reduced ensemble sizes, leaving only terminal sites at very dilute Pd concentrations.

These results appear counter to much of what we consider to be known about alloy catalysis—that is, it is the average electronic structure that is crucial for reactivity. Norskov et al. relate reactivity to the position and width of the d-band maximum,^46−48^ and their conclusions appear quite reasonable for a number of systems. In the case here, however, the electronic structure of these Cu–Pd alloys is considerably modified, as shown by valence-band studies, for instance.^1−7^ Thus, by the arguments of Norskov et al., and of many others, the stability of intermediates and the reactivity pattern *must* be significantly altered in a homogeneous manner relating to the d-band maximum position, yet apparently, they are not, and the effects strongly depend on the nature of binding of the adsorbate itself and the nature of the metals involved, as described above. How are we to view this then? Is it possible that the electronic structure, as revealed by ultraviolet photoelectron spectroscopy (UPS) and valence-band measurements, does not give a good representation of the surface with respect to reactivity? The answer to the latter question must be, at least in this instance, *yes*.

So, returning to the questions posed in the introduction, namely, (i) does alloying produce a homogeneous surface of quite different reactivity from the individual atoms, or (ii) a surface for which adjacent atoms are electronically modified by the second alloying metal or (iii) is the reactivity just a mix of the two properties? Clearly (i) is eliminated, and (iii) is the main observation here; that is, both monometallic Cu and Pd atom reactive characteristics are observed. However, considering (ii), their reactivity is indeed modified somewhat by the presence of the other metal, but this could either be due to electronic modification or, we feel more likely, due to the fact that the bonded state or transition state has to bond to both species during the decomposition process to form products. We propose that the ensemble effect is the dominant factor in the reactivity of CuPd.

## Supporting Information Available

More detailed information about the equipment used for the experiments (section 1), sample preparation (section 2, Figures) and the molecular beam system and methodology (section 3) (PDF)
