# Supplementary material for: The Catalytic Reactivity of Alloys; Ethanol and Formic Acid Decomposition on Cu–Pd(110)
Source: J Phys Chem C Nanomater Interfaces. 2022 Sep 12;126(37):15703–9. doi: 10.1021/acs.jpcc.2c04881 (PMC9511558; doi:10.1021/acs.jpcc.2c04881)
Supplement: Supplementary file 1 — jp2c04881_si_001.pdf [file jp2c04881_si_001.pdf]

Supporting Information for

The Catalytic Reactivity of Alloys; ethanol and formic acid decomposition on Cu-Pd(110)

Michael Bowker[1,2], Richard Holroyd[3], Neil Perkins[3,4]

[1]Catalysis Hub, RCAH, Rutherford Appleton Laboratory, Harwell Oxford, Didcot OX11 0QX, UK.

[2]Max Planck- Cardiff Centre on the Fundamentals of Heterogeneous Catalysis FUNCAT, Cardiff Catalysis Institute, School of Chemistry, Cardiff University, Main Building, Park Place, Cardiff, CF10 3AT, United Kingdom.

[3]Chemistry Dept., University of Reading, Reading, RG6 6AH

[4]now at Element Six, Campus, Harwell, Fermi Ave, Didcot OX11 0QR

1. UHV equipment.

This UHV machine used (fig. S1) was equipped with LEED, Auger electron spectroscopy and a mass spectrometer, as well as with the molecular beam, described in more detail below. It comprises a stainless steel UHV chamber maintaining a base pressure of ~ 2 x 10^{-10} mbar (95% H_2). It consists of the molecular beam itself, which delivers a circular beam at the sample of 2.9 mm. diameter with a flux of around 1.5 x 10^{17} molecules m^{-2} s^{-1}, or about 0.015 monolayers of molecules s^{-1} with respect to the number of surface atoms. The CuPd(110) crystal was mounted on a custom-built holder in the chamber centre; the preparation of the crystal is described below. The sample temperature was measured with a chromel-alumel thermocouple attached to the side of the crystal. In the same horizontal plane as the molecular beam enclosure, there was a rear-view retarding field analyser system with a coaxial electron gun (supplied by VG Microtech) for low energy electron diffraction (LEED) and Auger electron spectroscopy (AES). Also in the main UHV chamber was a quadrupole mass spectrometer (QMS) (Hiden Analytical Limited) employed for residual gas analysis (RGA). The QMS provides an indication of the angle-integrated partial pressure within the vacuum chamber, since no direct line of sight exists between the sample and detector.

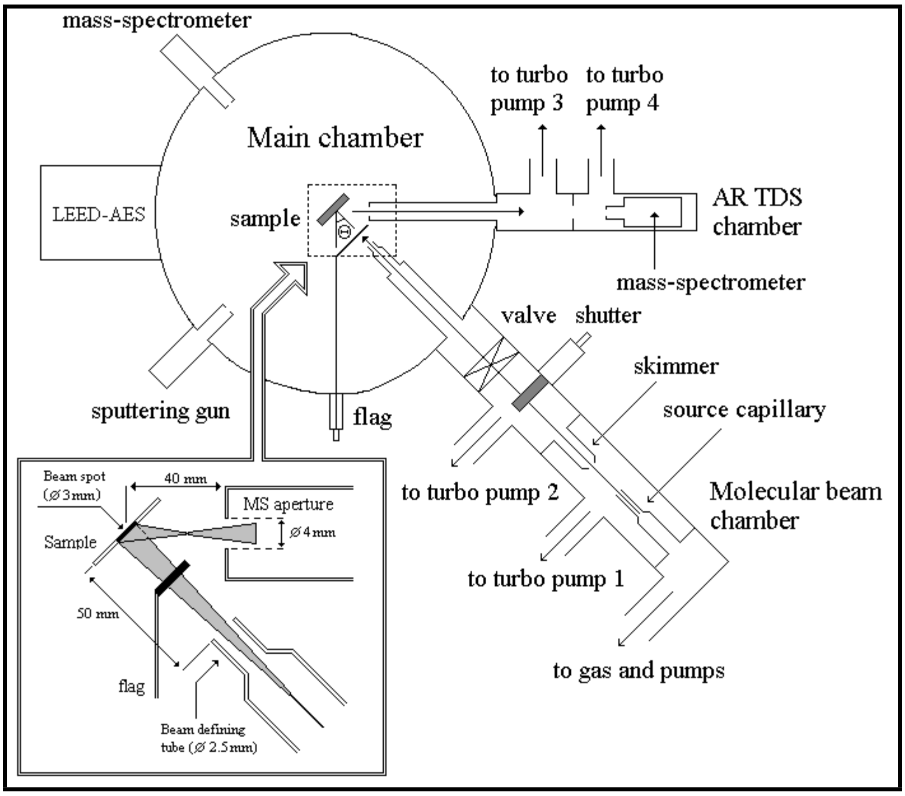

Figure S1. Schematic diagram of the UHV system.

2. **Sample preparation.** The initial cleaning consisted of cycles of Ar ion sputtering (2-3μA cm^{-2}), followed by annealing at a maximum temperature of 750K, after which a bcc (1x1) LEED pattern was obtained. Subsequently, by varying the sputtering and annealing temperatures and time periods various TL (top layer) compositions could be achieved, monitored through AES and LEED. The AES analysis was performed using Auger lines for Cu (66 eV) and Pd (333 eV). The escape depths were calculated to be respectively 4.9 and 6.5 Angstroms and was averaged here to 5.7 Angstroms to describe the SR (surface region) defined as the TL and three subsurface layers. The electron energies are close to the minimum in the escape depth dependence on electron energy, and thus provide a surface sensitive method for monitoring the SR.

The history dependence of the SR composition resulted in a specific treatment of the alloy to create the desired surface. Figure S2 shows the variation of SR concentration as a function of treatment.

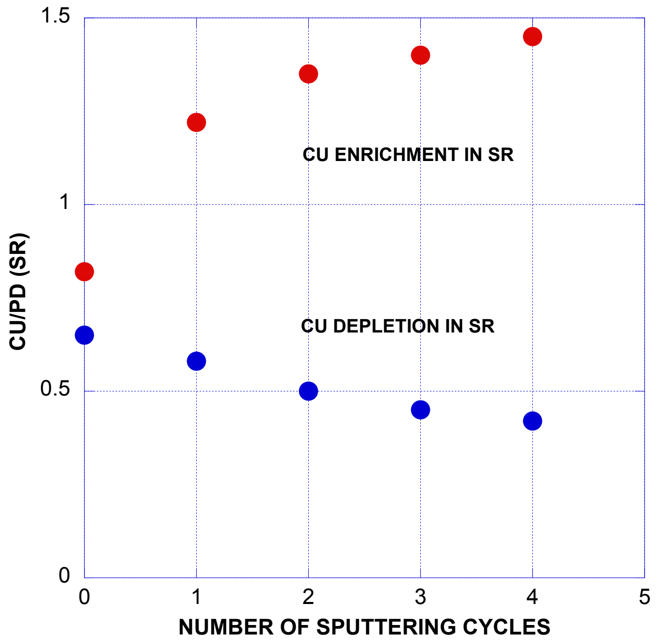

Figure S2. The effect of sputtering at two temperatures on the surface region Cu:Pd ratios. Blue circles 433K, red circles 713K.

The TL composition could be assessed by CO adsorption and then by monitoring the desorption, as described in detail by Mousa et al[1] in order to obtain the β-1:1 Cu:Pd TL composition. The desorption of CO from Cu(110) is at very low temperature (~200K)[2,3], from Pd(110) it is at very high temperature (~450-500K)[4,5], while the maximum peak temperature for a well mixed CuPd layer is around 380K[1] and as also can be seen in the main text fig. 3b.

3. **Molecular beam system.** The thermal, nozzle source molecular beam system has been described in detail previously[6-8]. The beam housing comprises two stainless steel six way cross junctions and one four way cross junction (70 mm o.d. flanges). The four way cross is backed by an Edwards rotary pump and acts as a source chamber. One six way cross acts as a beam production chamber, the other six way junction defines the beam. Both six way crosses are backed by Leybold turbomolecular pumps, which in turn are differentially backed by two Edwards rotary pumps. The entire beam housing is bolted to the main chamber via a fast acting gate valve, allowing either region to be isolated from the other. Gases entering the source chamber from the gas line were at a pressure of 20 mbar in all experiments. Gas can be stored in two glass reservoirs (5L capacity) which also act as ballasts in the gas line. This gas effuses through a quartz capillary (nozzle) mounted at the end of the source chamber. The xyz position of the capillary can be manipulated to maximise beam alignment and flux in the analysis chamber using mass spectrometry and argon gas. The beam production chamber

contains a conical skimmer with a central hole between the two six way junction crosses. This collimates the beam, removing out-of-axis scattered molecules. The beam definition stage contains a 'beam flag' which can block the beam path in an instantaneously. This flag can be switched 'on' and 'off' manually, or by PC-controlled motors. Also, in this stage a Pyrex tube acts as the final beam collimator. Its exit is 50 mm from the centre of the analysis chamber and the crystal sample. An ion gauge is also placed in this section to measure the pressure in this region. The effective pressure of the gases at the crystal surface (20 mbar source) is thus between 10^{-7} and 10^{-8} mbar (for example with Ar it is 3.5×10^{-8} mbar). In the main chamber, between the crystal surface and the Pyrex tube, is a 'chamber flag' which can block the beam directly in front of the crystal face. It is controlled in the same way as the beam flag and permits measurement of the sticking probability of incident beams in terms of pressure, and its coverage dependence.

The adsorption characteristics are measured by analysing the reflected gases from the surface and the methodology is that described below[6-8]. The sticking probability is determined from the amount of gas adsorbed (that is that not reflected from the surface), and the total reflection determined from a non-adsorbing surface.

References

1. Mousa, M.S.; Laboda-Cackovic, J.; Block, J.H., Characterization of PdCu (110) single crystal surface compositions during CO chemisorption, Vacuum, 1995, 46, 117.
2. Harendt, C.; Goschnick, J; Hirchwald, W., Surf. Sci., 1985, 152/3, 453
3. Horn, K.; Hussain, M.; Pritchard, J., Surf. Sci., 1977, 63, 244.
4. He, J-W.; Norton, P.R., J. Chem. Phys., 1988, 89, 1170.
5. Jones, I.; Bennett, R. A.; Bowker, M., CO Oxidation on Pd(110): A high Resolution XPS and Molecular Beam Study, Surface Sci., 1999, 439, 235-48.
6. Bowker, M. The molecular beam reactor, Appl. Catal. A: General 1997,160, 89.
7. King, D.A.; Wells M. G. Molecular beam investigation of adsorption kinetics on bulk metal targets: Nitrogen on tungsten, Surf. Sci., 1971, 29, 454.
8. Bowker, M.; King, D. A. Oxygen diffusion on tungsten single crystal surfaces: Secondary electron emission studies, Surf. Sci., 1980, 94, 564.
